# Retraction Note to: MiR-143-3p functions as a tumor suppressor by regulating cell proliferation, invasion and epithelial–mesenchymal transition by targeting QKI-5 in esophageal squamous cell carcinoma

**DOI:** 10.1186/s12943-022-01639-0

**Published:** 2022-08-24

**Authors:** Zhenyue He, Jun Yi, Xiaolong Liu, Jing Chen, Siqi Han, Li Jin, Longbang Chen, Haizhu Song

**Affiliations:** 1 Department of Medical Oncology, Jinling Hospital, Medical School of Nanjing University, 305 Zhongshan East Road, Nanjing, Jiangsu 210002 People’s Republic of China; 2 Department of Cardiothoracic Surgery, Jinling Hospital, Medical School of Nanjing University, 305 Zhongshan East Road, Nanjing, Jiangsu 210002 People’s Republic of China

**Retraction note to: Mol Cancer 15, 51 (2016)**

**https://doi.org/10.1186/s12943-016-0533-3**

The Editor in Chief has retracted this article because of significant concerns regarding a number of Figures presented in this work, which question the integrity of the data. The Editor-in-Chief therefore no longer has confidence in the integrity of the data in this article. All authors agree to this retraction.

